# Exercise training augments brain function and reduces pain perception in adults with chronic pain: A systematic review of intervention studies

**DOI:** 10.1016/j.ynpai.2023.100129

**Published:** 2023-04-20

**Authors:** Kierstyn L. Palmer, Madeline E. Shivgulam, Anne Sophie Champod, Brian C. Wilson, Myles W. O'Brien, Nick W. Bray

**Affiliations:** a School of Health and Exercise Sciences, University of British Columbia Okanagan, Kelowna, V1V 1V7, Canada; b Division of Kinesiology, Dalhousie University, Halifax, Nova Scotia, B3H 3J5, Canada; c Dept. of Psychology, Acadia University, Wolfville, Nova Scotia, B4P 2R6, Canada; d Department of Biology, Acadia University, Wolfville, Nova Scotia, B4P 2R6, Canada; e School of Physiotherapy (Faculty of Health) and Department of Medicine, Dalhousie University, Halifax, Nova Scotia, B3H 3J5, Canada; f Cumming School of Medicine, Dept. of Physiology & Pharmacology, University of Calgary, Calgary, Alberta, T2N 4N1, Canada; g Hotchkiss Brain Institute, University of Calgary, Calgary, Alberta, T2N 1N4, Canada

**Keywords:** Physical activity, Pain inhibition, Functional neuroimaging, Subjective pain measurement

## Abstract

**Introduction:**

Chronic pain (CP) is a leading cause of disability worldwide. Pain may be measured using subjective questionnaires, but understanding the underlying physiology, such as brain function, could improve prognosis. Further, there has been a shift towards cost-effective lifestyle modification for the management of CP.

**Methods:**

We conducted a systematic review (Registration: #CRD42022331870) using articles retrieved from four databases (Pubmed, EMBASE, AMED, and CINAHL) to assess the effect of exercise on brain function and pain perception/quality of life in adults with CP.

**Results:**

Our search yielded 1879 articles; after exclusion, ten were included in the final review. Study participants were diagnosed with either osteoarthritis or fibromyalgia. However, two studies included “fibromyalgia and low back pain” or “fibromyalgia, back, and complex regional pain.” Exercise interventions that were 12 weeks or longer (n = 8/10) altered brain function and improved pain and/or quality of life outcomes. The cortico-limbic pathway, default-mode network, and dorsolateral prefrontal cortex were key regions that experienced alterations post-intervention. All studies that reported an improvement in brain function also demonstrated an improvement in pain perception and/or quality of life.

**Discussion:**

Our review suggests that alterations in brain function, notably the cortico-limbic, default-mode and dorsolateral prefrontal cortex, may be responsible for the downstream improvements in the subjective experience of CP. Through appropriate programming (i.e., length of intervention), exercise may represent a viable option to manage CP via its positive influence on brain health.

## Highlights

• Chronic pain (CP) is pain that is experienced for longer than three months.
• Exercise interventions longer than 12 weeks improve brain function and pain perception in individuals suffering from CP.
• The cortico-limbic, default mode, and dorsolateral prefrontal cortex appear to be critical brain regions.
• Changes in brain function may be responsible for improvements in the subjective experience of CP post-exercise intervention.
• Exercise may represent a cost-effective therapy for managing CP via its positive influence on brain function.

## 1 Introduction

Pain is an unpleasant sensory and emotional experience associated with potential or actual tissue damage (Raja et al., 2020). If pain is experienced for three months or more, regardless of location, it is considered *chronic pain (CP)*, a leading cause of disability worldwide (QuickStats, 2016). Although the mechanisms governing CP are complex and not yet fully understood, it is thought that some types of CP result from disruption of pain inhibitory pathways (Julien et al., 2005), leading to altered pain processing (Staud et al., 2008). Further, perceived pain levels can be intensified through increased responsiveness of the nervous system to harm, otherwise known as central sensitization (Bingel and Tracey, 2008, Yang and Chang, 2019 Jun 26). Several variables also influence pain perception, including but not limited to environment, emotions, genetics, age, and sex (Bingel and Tracey, 2008, Yang and Chang, 2019 Jun 26). Understanding the underlying physiology and factors that contribute to CP could improve its prognosis and management.

Pain intensity may be measured using questionnaires. Such questionnaires can be specific to the type of CP, measure the impact of CP on quality of life, and/or measure general pain intensity (Dworkin et al., 2005, Camann, 1999, von Baeyer et al., 2009, Larsson et al., 2015, Kong et al., 2019, Tüzün et al., 2005, Price et al., 1983). These questionnaires are inherently prone to bias caused by patients' attitudes (including thoughts and emotions) and interviewer predisposition (Xu and Huang, 2020). Due to the subjective nature of pain, a truly objective measure of CP is not currently possible. However, utilizing a physiological measurement may provide greater insight while avoiding the inherent biases of questionnaires (Xu and Huang, 2020). Functional magnetic resonance imaging (fMRI) and functional near-infrared spectroscopy (fNIRS) are two neuroimaging tools that can be used to objectively measure “brain function” via cerebral oxygenation (Xu and Huang, 2020, Karim et al., 2012); for the purpose of this review, brain function refers to both brain activity and connectivity. Indeed, people with CP demonstrate altered brain function when compared to healthy controls (Baliki and Apkarian, 2015, Heitmann et al., 2022); this reflects the *plastic* nature of the brain or its ability to functionally and structurally change with repeated exposure to (pain-inducing) stimuli (Mateos-Aparicio and Rodríguez-Moreno, 2019). In addition to providing greater insight into the mechanisms of pain processes, fMRI and fNIRS can be used to evaluate the efficacy of and understand the underlying physiological changes induced by CP interventions.

Opioid medications are a widely used CP treatment (Wang et al., 2019). However, they are highly addictive and, as a result, have led to an epidemic of opioid addiction and overdoses (Wang et al., 2019); since the 1990s, the number of deaths due to opioid-induced overdoses has quadrupled (US Department of Health and Human Services, 2021). Conversely, physical exercise (e.g., aerobic, resistance training, Tai Chi, etc.) has demonstrated many benefits in those suffering from CP, including: increasing mood-enhancing chemicals, promoting pleasure/reward circuitry in the brain, improving quality of life, and reducing perceived levels of pain, disability, and pain-induced anxiety (Polaski et al., 2021). Such changes are in addition to a multitude of other benefits that extend beyond pain, and is exactly why exercise has been described as having a *sledgehammer effect* on human health (Erickson et al., 2022). Ultimately, exercise may be a viable intervention strategy to promote pain management in those suffering from CP.

To our knowledge, only one systematic review has been conducted on the effects of exercise on fMRI-determined brain function in individuals with CP (de Zoete et al., 2020). While exercise promoted functional brain changes, there was heterogeneity within brain regions studied and exercise strategies used across studies (de Zoete et al., 2020). Further, this review focused specifically on (chronic) musculoskeletal pain and, as a result, included only four studies (de Zoete et al., 2020). Therefore, the purpose of our systematic review was to investigate the effect of exercise on brain function (as assessed by fMRI and fNIRS) and measures of pain perception and quality of life in adults living with CP. We hypothesized that exercise would improve: 1) brain function; and 2) pain perception and quality of life in those suffering from CP.

## 2 Methods

### 2.1 Study design and search strategy

This systematic review was registered with PROSPERO (Registration number: CRD42022331870) (Supplemental Material A) and followed the Preferred Reporting Items for Systematic Review and meta-Analysis (PRISMA) statement (Moher et al., 2009). Similar to previous systematic reviews conducted by our group (Bray et al., 2021, Champod et al., 2018, O’Brien et al., 2022) and others in the field (de Zoete et al., 2020), we included the following databases: PubMed, EMBASE, AMED, and CINAHL. The search strategy was developed based on current guidelines for the design of systematic reviews (Selcuk, 2019), our prior experience with systematic reviews, and input from content experts (Belavy et al., 2021). The search strategy consisted of related terms for “exercise,” “functional neuroimaging,” and “pain” (Supplemental Material B); terms were searched in each database as title and abstract keywords, as well as subject headings (Supplemental Material C). There were no restrictions on country, language, or publication period. The search included articles from inception to March 4th, 2023.

### 2.2 Screening and inclusion criteria

Screening was completed in two steps using Covidence (Veritas Innovation ltd, Australia): 1) title and abstract; and 2) full-text. Studies were independently screened by two authors (KLP and MES). Following each step, reviewers met to discuss any inconsistencies regarding their inclusion decisions. If a resolution was not found, a senior reviewer (NWB) was consulted to make a final decision.

Studies were included if they: 1) conducted a repetitive (i.e., greater than one session) intervention study with experimental manipulation of physical exercise (Caspersen et al., 1985); 2) measured brain function via fMRI and/or fNIRS under any conditions (i.e., rest, task, etc.); 3) measured pain perception and/or quality of life via questionnaires or other subjective measures, such as sensitivity tests (e.g., Visual Analogue Scale, Numerical Rating Scale, pressure algometer); and 4) included human participants ≥18 years of age who were experiencing CP, but otherwise healthy. Finally, back-searching was conducted on included studies to ensure that no additional studies satisfied the inclusion criteria.

### 2.3 Bias assessment

The quality of individual studies was independently assessed by two reviewers (KLP and MES), and any inconsistencies were resolved by a third (NWB). We utilized the National Institutes of Health bias assessments for 1) controlled intervention studies; and 2) before-after (pre-post) studies with no control group (National Institutes of Health, 2021). As per the tool creators, specific quality assessment scores were not calculated. As a result, no studies were excluded based on quality assessment scores.

### 2.4 Outcome measures

The primary outcome measures were: 1) brain function, as measured by fMRI and/or fNIRS; and 2) pain perception and/or quality of life scores. Notably, brain function was separated into two categories: 1) brain activity, which subsequently permits the measurement of 2) functional brain connectivity or regions of the brain that are anatomically separate but temporally synchronized in their activation (Damoiseaux et al., 2008). Reporting of these outcomes varied between individual studies, as many focused on widespread regions of interest.

### 2.5 Data extraction

We extracted the following data from all included studies: 1) authors and publication date; 2) participant characteristics (age, sex, CP diagnosis); 3) details of the physical exercise intervention (frequency, intensity, type, time, volume, and progression); 4) change in outcome measurements (e.g., brain activity or functional brain connectivity, pain perception and/or quality of life) and 5) method of collection (i.e., imaging and questionnaire type). No studies were excluded from this review based on quality assessment, although quality assessment was used to provide context when interpreting the study findings.

## 3 Results

### 3.1 Search results

**Fig. 1:**
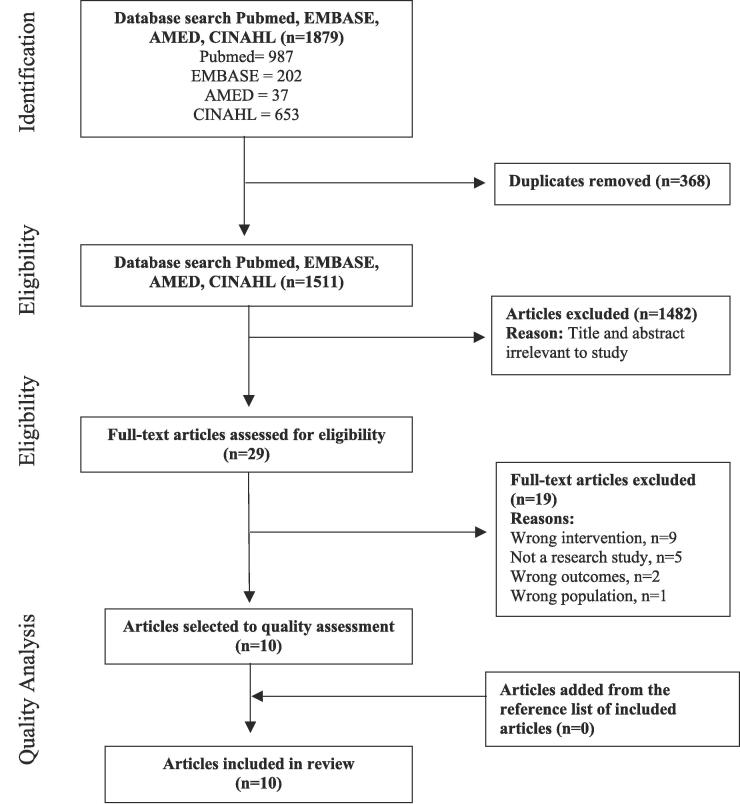
PRISMA flow diagram of study selection and quality analysis.

1879 articles were identified via our electronic search. 1511 studies remained after duplicates were removed (n = 368), and 29 articles were retained for full-text screening. No additional articles were identified via back searching, leaving ten articles to be included in the present review (Fig. 1) (Flodin et al., 2015, Kong et al., 2021, Liu et al., 2019, Liu et al., 2019, Martinsen et al., 2018, Micalos et al., 2014, Shen et al., 2021, Ozturk et al., 2021, Lofgren et al., 2023, de Winckel et al., 2022).

### 3.2 Quality assessment

**Fig. 2:**
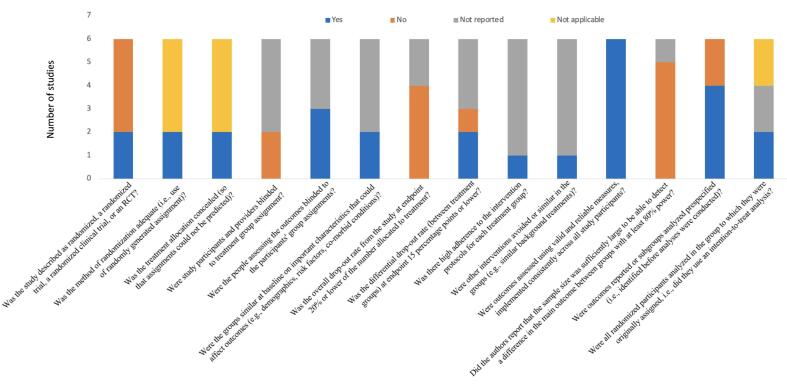
Quality assessment summary of studies with a control group. See Supplemental Material D for more details.

**Fig. 3:**
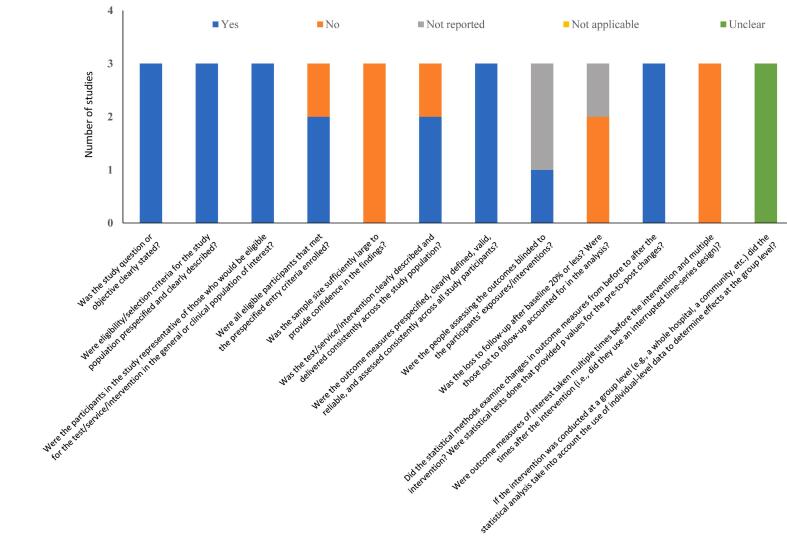
Quality assessment summary of studies with no control group. See Supplemental Material E for more details.

“No” and “not reported” were the most frequent answers for two studies (Flodin et al., 2015, Micalos et al., 2014), suggesting poor or low quality; the remaining studies were of relatively higher quality (Liu et al., 2019, Liu et al., 2019, Martinsen et al., 2018, Shen et al., 2021, Ozturk et al., 2021, Lofgren et al., 2023, de Winckel et al., 2022) (Fig. 2, Fig. 3). Broadly, studies with a control group poorly reported or did not report the blinding of participants and providers, adherence to intervention protocols, and if the sample size was large enough to detect differences between groups (Flodin et al., 2015, Kong et al., 2021, Martinsen et al., 2018, Micalos et al., 2014). Conversely, they implemented valid and reliable outcome measurements across study participants (Flodin et al., 2015, Kong et al., 2021, Martinsen et al., 2018, Micalos et al., 2014). Broadly, studies without a control group poorly reported or did not report if the sample size was large enough to detect differences between groups and what participants were lost to follow-up (Liu et al., 2019, Liu et al., 2019, Shen et al., 2021, Ozturk et al., 2021). Conversely, they did well in all other quality assessment questions, including a clear statement of study objective(s), eligibility criteria, reliable outcome measurements, and the use of statistical methods to examine pre- and post-intervention changes (Liu et al., 2019, Liu et al., 2019, Shen et al., 2021, Ozturk et al., 2021, Lofgren et al., 2023, de Winckel et al., 2022). The detailed quality assessment is available in Supplementary Materials D & E. Importantly, two studies were produced from the same trial, which means the results include identical participants, despite focusing on different brain regions of interest (Liu et al., 2019, Liu et al., 2019).

### 3.3 Population demographics and study design characteristics

The ten included studies focused on patients diagnosed with osteoarthritis (n = 4) (Liu et al., 2019, Liu et al., 2019, Shen et al., 2021, Ozturk et al., 2021), fibromyalgia (n = 4) (Flodin et al., 2015, Kong et al., 2021, Martinsen et al., 2018, Lofgren et al., 2023), or multiple types of CP, including “fibromyalgia and back pain” (n = 1) (de Winckel et al., 2022) and “fibromyalgia, back, and complex regional pain” (n = 1) (Micalos et al., 2014) (Table 1). The mean age of participants ranged from 42 to 65 years, although two studies simply reported a range (40–70 years) (Liu et al., 2019, Liu et al., 2019), and one study had an average median of 53.5 (Lofgren et al., 2023). Altogether, there were more female than male participants (398/488 total participants). Most studies' sample size was less than 50 (Liu et al., 2019, Liu et al., 2019, Shen et al., 2021, Ozturk et al., 2021, Lofgren et al., 2023, de Winckel et al., 2022), but the two studies from the same trial had the largest sample size (n = 140) (Liu et al., 2019, Liu et al., 2019).

**Table 1:** Characteristics of included studies.

**Study ID** **(Author, Year)**	**Sample size (male)**	**Average age (mean ± SD)**	**Diagnosis**	**Exercise Modality**	**Connectivity/Activity**	**Pain**	**QL**
**Flodin et al.** (2015)	38 (0)	I: 48.4 C: 41.8	Fibromyalgia	Resistance training	↑	NC	Improved
**Kong et al.** (2021)	48 (NR)	I: 51.6 ± 11.6 C: 52.3 ± 10.4	Fibromyalgia	Tai Chi	↑	Improved	Improved
**Liu et al.** (Liu et al., 2019, Liu et al., 2019)	140 (25)	40–70	Osteoarthritis	Baduanjin, Tai Chi, Aerobic training	↑↓	Improved	Improved
**Lofgren et al.** (2023)	122 (0)	I: 51 (median) C: 56 (median)	Fibromyalgia	Resistance Training	↑	Improved	Improved
**Shen et al.** (2021)	17 (0)	64.5 ± 6.7	Osteoarthritis	Tai Chi	NC	Improved	Improved
**Lofgren et al. (**2023**)**	122 (0)	I: 51 (median) C: 56 (median)	Fibromyalgia	Resistance Training	↑	Improved	Improved
**Martinsen et al.** (2018)	31 (0)	49.6	Fibromyalgia	Resistance training	↑	Improved	Improved
**Micalos et al.** (2014)	19 (3)	I: 50 ± 12 C: 49.6 ± 10	Fibromyalgia Back Regional	Aerobic training	NC^§^	NC^§^	NR^§^
**Ozturk et al.** (2021)	15 (1)	59.2 ± 6.28	Osteoarthritis	Resistance and Flexibility training	↓^§^	Improved^§^	Improved^§^
**Van de Winckel et al.** (2022)	58 (13)	I1: 46.43 ± 14.28 I2: 44.88 ± 15.68 C: 39 ± 16.45	Chronic Low Back Pain	Qiqong and Resistance Training	↑	Improved	Improved

Note: Lofgren is reported twice because it measured activity and connectivity. SD = standard deviation, QL = quality of life, I = intervention, C = control, NC = no significant change, NR = not reported, § = outcome was measured in conjunction with pain stimulus.

Most studies focused on one exercise modality (n = 7), including resistance training (n = 4), Tai Chi (n = 2), and aerobic training (n = 1). Three studies incorporated multi-domain intervention strategies, including multiple exercise modalities (Tai Chi, Buandjin, aerobic training or qigong and resistance training) and health education (Liu et al., 2019, Liu et al., 2019, de Winckel et al., 2022) (Table 2). Across all studies (multimodal and non-multimodal), Tai Chi (n = 4) and resistance training (n = 4) were the most reported exercise modality, followed by aerobic training (n = 3). Interventions ranged from 6 to 15 weeks in duration, however, eight studies had interventions of 12 weeks or more (Lofgren et al., 2023, de Winckel et al., 2022, Flodin et al., 2015, Kong et al., 2021, Liu et al., 2019, Liu et al., 2019, Martinsen et al., 2018, Micalos et al., 2014). Healthy (non-CP) individuals were included as controls in six studies (n = 4) (Flodin et al., 2015, Kong et al., 2021, Martinsen et al., 2018, Micalos et al., 2014, Lofgren et al., 2023, de Winckel et al., 2022). Alternatively, the other four studies did not include a control group (Liu et al., 2019, Liu et al., 2019, Shen et al., 2021, Ozturk et al., 2021).

**Table 2:** Exercise parameters of included studies.

**Study ID** **(Author, Year)**	**FITTVP**					
	**Frequency**	**Intensity**	**Time** **(minutes)**	**Type**	**Volume (minutes)**	**Progression**
**Flodin et al. (**2015**)**	2x/week × 15 weeks	40 % of one 1 RM 15–20 reps for 1–3 sets	60	Resistance Training	120	Week 5: 50 % x1RM 2 sets of 12–15 reps. Week 8: 60 % x 1RM 2 sets of 10–12 reps Week 12: 70 % x 1RM 2 sets of 8–10 reps
**Kong et al.** (2021)	2x/week × 12 weeks + 30 min practice × day	Not reported	60	Tai Chi	120	Not reported
**Liu et al.** (Liu et al., 2019, Liu et al., 2019)	5x/week × 12 weeks	Not reported	60	Tai Chi, Badjuanjin, Aerobic Training	300	Not reported
**Lofgren et al.** (2023)	2x/week × 15 weeks	40 % of one 1 RM 15–20 reps for 2 sets	50	Resistance Training	100	Up to 80 % of 1RM with 5–8 reps
**Shen et al.** (2021)	3x/week × 8 weeks	Not reported	60	Tai Chi	180	Not reported
**Martinsen et al.** (2018)	2x/week × 15 weeks	Not reported	60	Resistance Training	120	Not reported
**Micalos et al.** (2014)	2x/week × 12 weeks	Not reported	20	Aerobic Training	40	Not reported
**Ozturk et al.** (2021)	3x/week × 6 weeks	30–60 % of 10 RM, for 10 reps	45	Resistance and Flexibility Training	135	Week 3: + 1 set of 3 reps
**Van de Winckel et al.** (2022)	3x/week × 12 weeks	Not reported	41	Qiqong and Resistance Training	123	Not reported

Note: RM = rep maximum, Volume = Frequency × days per week.

### 3.4 Brain function

Of the eight included studies, six measured functional brain connectivity (Shen et al., 2021, Lofgren et al., 2023, Flodin et al., 2015, Kong et al., 2021, Liu et al., 2019, Liu et al., 2019). Three recorded increases (Flodin et al., 2015, Kong et al., 2021, Lofgren et al., 2023) and two recorded bidirectional changes (Liu et al., 2019, Liu et al., 2019), respectively; notably, the bidirectional changes were from studies that used the same cohort. One study did not observe a significant change in functional brain connectivity (Shen et al., 2021), but it contained the lowest sample size (n = 17), included no control group, and was the only intervention less than 12 weeks to measure connectivity (Shen et al., 2021). Across the six studies, the anterior cingulate cortex (n = 4), medial prefrontal cortex (n = 4), posterior cingulate cortex (n = 2), thalamus (n = 2), and cerebellum (n = 2) increased connectivity with a multitude of regions, but most commonly, the hypothalamus, dorsolateral prefrontal cortex (DLPFC), periaqueductal gray, and ventral tegmental area (Shen et al., 2021, Flodin et al., 2015, Kong et al., 2021, Liu et al., 2019, Liu et al., 2019). For the two studies that demonstrated bidirectional change, the DLPFC, as well as the ventral tegmental and periaqueductal gray regions, decreased connectivity with several other areas (Liu et al., 2019, Liu et al., 2019) (Supplemental Material F).

Four studies focused on changes in (whole brain) activity (Martinsen et al., 2018, Micalos et al., 2014, Ozturk et al., 2021, de Winckel et al., 2022) (Supplementary Material G). Importantly, three of these studies measured brain function during the application of pressure (pain) stimuli (Micalos et al., 2014, Ozturk et al., 2021) and one was also the only study to utilize fNIRS in measuring brain function (Ozturk et al., 2021). One did not demonstrate a change in activity (Micalos et al., 2014), while the other demonstrated a decrease in activity of the DLPFC (Ozturk et al., 2021). The other two studies measured brain activity without the application of pain stimuli and demonstrated increased activation in the amygdala, cerebellum, and putamen (Martinsen et al., 2018), as well as the parietal operculum, angular gyrus, precentral gyrus, and supramarginal gyrus (de Winckel et al., 2022).

### 3.5 Pain perception and quality of life

Studies measured pain perception and/or quality of life outcomes utilizing the Short-Form Health Survey (n = 3), Visual Analogue Scale (n = 4), McGill Pain Questionnaire (n = 1), Fibromyalgia Impact Questionnaire (n = 4), Knee Osteoarthritis Outcome Scale (n = 2), Western Ontario and McMaster Universities Osteoarthritis Index (n = 2), Beck Depression Inventory (n = 2), and the Hospital Anxiety and Depression Score (n = 2). All but one study (Micalos et al., 2014) reported improvements post-exercise in the measure of pain perception and/or quality of life; however, this particular study was the only one to include multiple types of CP, including fibromyalgia, back pain, and complex regional pain, and recorded brain function during the application of pressure (pain) stimulation (Micalos et al., 2014) (Supplementary Material H). Importantly, people with CP experienced an improvement in pain perception and/or quality of life in all studies that recorded a change in brain function and conducted their exercise intervention for 12 weeks or longer (n = 8/10) (Flodin et al., 2015, Kong et al., 2021, Liu et al., 2019, Liu et al., 2019, Martinsen et al., 2018, Ozturk et al., 2021, Lofgren et al., 2023, de Winckel et al., 2022).

## 4 Discussion

We conducted a systematic review to assess the effect of exercise interventions on brain function (connectivity and activity) and pain perception/quality of life in adults with CP. In partial support of our hypothesis, exercise improved brain function in all studies that conducted a 12+ week intervention and measured brain function in the absence of pain stimulation. Also in partial support of our hypothesis, all studies that reported an improvement in brain function after 12 weeks or more of exercise also demonstrated an improvement in pain perception and/or quality of life. Such findings suggest that exercise-induced improvements in CP may be mediated through changes in brain function but that the parameters (i.e., frequency, intensity, etc.) of the intervention are critical.

### 4.1 Brain function measures and implications

*Cotico-limbic:* The anterior cingulate cortex, amygdala, thalamus, and medial prefrontal cortex reported increased connectivity with various regions of interest across multiple studies (Micalos et al., 2014, Damoiseaux et al., 2008, Flodin et al., 2015, Kong et al., 2021, Liu et al., 2019); although conflicting research exists, increases in connectivity between regions linked or part of an identified network are believed to reflect improved brain health. The amygdala and thalamus were also regions of interest in studies focused on whole-brain activity (Martinsen et al., 2018, Micalos et al., 2014). Together, the anterior cingulate cortex, amygdala, thalamus, and medial prefrontal cortex play key roles in the cortico-limbic system, a pathway of regions that have been implicated during pain processing (Rusbridge, 2020). More specifically, the cortico-limbic system encompasses the flow of information from higher cortical brain areas to the spinal level, which dictates emotional and behavioural responses to pain (Yang and Chang, 2019). Therefore, this system plays a critical function in pain regulation, as it has the ability to inhibit pain by exerting top-down pain control on the descending pain modulation system (Yang and Chang, 2019 Jun 26, Rusbridge, 2020).

It is hypothesized that the maladaptive functioning of the cortico-limbic system, that is, neural reorganization as a result of prolonged exposure (i.e., chronic) to pain (Yang and Chang, 2019 Jun 26, Rusbridge, 2020), may maintain CP. The persistence of pain in people with CP is likely attributable to impaired functioning or reduced connectivity between regions of the cortico-limbic pathway that are responsible for pain inhibition/control. Other research supports this hypothesis, demonstrating reduced connectivity between several parts of the cortico-limbic system, including the hypothalamus, thalamus, amygdala, and the medial prefrontal cortex in people with CP (Kong et al., 2021, Ayoub et al., 2019). Relative to the present review, the observed increases in connectivity between cortico-limbic brain regions suggest how exercise may help to normalize or restore the functioning of the cortico-limbic pathway. Such findings are supported further by the consistent downstream improvements in subjective pain perception and/or quality of life measures.

*Default Mode Network:* Despite being a part of the cortico-limbic pathway, the medial prefrontal cortex and posterior cingulate cortex are also regions belonging to a functional network known as the default-mode (Raichle et al., 2001); this highlights a key point, that is, a single region can belong to multiple networks or pathways and our understanding of functional brain connectivity is still developing. The default mode network is upregulated when a person is engaged in mind-wandering or passive tasks, such as thinking about the past or dreaming about the future (Anticevic et al., 2012). For this reason, it also plays a role in memory and is known to compete with attention-requiring processes (Raichle et al., 2001, Anticevic et al., 2012). Downregulation of the default mode network suggests that attention-requiring processes, such as those involved in the frontoparietal or executive function network are in use (Beaty et al., 2015). While a healthy individual may be “daydreaming” at rest, a patient with CP is more likely to be thinking or reminded about the pain they are experiencing and, therefore, less likely to be in a *default* state (Čeko et al., 2020, Alshelh et al., 2018). Alterations of the default mode network have been recorded in people with CP, specifically, decreased connectivity between known regions or hubs (Baliki and Apkarian, 2015, Čeko et al., 2020). The present review demonstrated that exercise increased connectivity between regions within and beyond the default mode network, including two of its key hubs, the medial prefrontal and posterior cingulate cortex. Increasing connectivity and, by extension, upregulation of the default mode network suggests a downregulation of attention-requiring processes; this may mean the CP patient is no longer focused or attentive to their pain and, instead, free to daydream like a pain-free individual. Again, such findings are supported by the consistent improvements in pain and quality of life outcomes for the included studies.

*DLPFC:* The DLPFC experienced bidirectional changes in connectivity with multiple brain regions. More specifically, the DLPFC increased connectivity with regions belonging to the cortico-limbic system (anterior cingulate cortex and medial prefrontal cortex) (Liu et al., 2019) and decreased connectivity with regions beyond the cortico-limbic system, such as the supplemental motor areas and the temporoparietal junction (Liu et al., 2019). At a minimum, such changes suggest that exercise can alter DLPFC connectivity in people with CP. Admittedly, it is difficult to draw more meaningful interpretations of the bidirectional changes observed in DLPFC connectivity. In considering the simultaneous changes in clinical outcomes, we theorize that increased connections between the DLPFC and the cortico-limbic pathway are *good*, while connections to other regions, which were decreased or downregulated within the present review, are *bad*.

Within the present review, the DLPFC was the only region to also report bidirectional changes in activity, specifically an increase (Lofgren et al., 2023) and decrease (Ozturk et al., 2021) during the application of pain stimuli. Other researchers have suggested that upregulated DLPFC activation is related to the production and continuation of CP (Liu et al., 2019). Past research has also demonstrated abnormally high DLPFC activity in people with CP (Weissman-Fogel et al., 2008), as well as increases in DLPFC activation during the application of noxious pressure stimuli (Ozturk et al., 2021). Further research supports the potential of treating CP via targeted regulation of DLPFC activity (Seminowicz and Moayedi, 2017). Taken together, such findings may suggest that downregulation of the DLPFC in CP may be associated with restoration or *normalization* of brain function and subsequent improvements in pain perception/quality of life in people with CP post-exercise.

### 4.2 Mechanisms

The mechanistic model suggests exercise induces behavioral or clinical change by acting on multiple physiological levels, including molecular, cellular, and structural/functional (El-Sayes et al., 2019). In summary, exercise induces upregulation of molecules that cause positive changes at the cellular level, including the formation of new neurons (i.e., neurogenesis and gliogenesis), connections between neurons (i.e., synaptogenesis), and blood cells (i.e., angiogenesis) (El-Sayes et al., 2019). Developing new cells with more synapses increases gray and white matter volume, which influences receptor activity and blood flow (El-Sayes et al., 2019). Exercise may also improve brain function and perceived pain scores in those suffering from CP via its anti-inflammatory effect (da Scheffer and Latini, 2020). The inflammatory response is designed to protect and heal the body from injury; however, increased or consistently high levels of inflammatory markers are present in people with prolonged pain, such as adults with CP (Morris et al., 2020). Exercise has been shown to decrease/increase levels of pro-/anti-inflammatory molecules within the nervous system (da Scheffer and Latini, 2020). Finally, exercise, particularly resistance training and Tai Chi, can exert a direct effect on the area or sources of pain by strengthening the bones, muscles, tendons, and ligaments (United Kingdom National Health Service, 2022). Future CP research is investigating the relationship between exercise, inflammation, and pain to understand what governs the relationship and, therefore, shape future CP treatments (Martin Ginis et al., 2020).

### 4.3 Previous research

A 2020 systematic review examined the effect of exercise on brain function in patients with strictly chronic musculoskeletal pain and reported changes post-intervention (de Zoete et al., 2020). However, the authors of the 2020 review did not report specific regions of interest. As such, it is difficult to draw more specific comparisons (de Zoete et al., 2020). An umbrella review encompassing 21 Cochrane Reviews with a total of 381 studies concluded that there was evidence of improved physical function but a variable effect on both psychological function and quality of life outcomes in people with CP post-exercise (Geneen et al., 2017). Another systematic review observed increased cerebral blood flow to the thalamus and anterior cingulate cortex, along with a reduction in pain intensity following other interventional strategies (Kim et al., 2021). Taken together, the present review and previous research suggest that 12 weeks or more of exercise alters brain health in people with CP and that the cortico-limbic, default-mode, and DLPFC are central players; such alterations are responsible for the (downstream) improvements in pain perception and life quality (Kim et al., 2021).

### 4.4 Limitations and future directions

We conducted the first systematic review on the effect of physical exercise on global CP, but it is not without limitations. Most notably, our review included just ten studies, and only two were randomized controlled trials. Admittedly, this was surprising given that CP is a leading cause of disability worldwide. The limited literature forced us to modify the original criteria of our PROSPERO document to include all types of intervention studies (randomized and non-randomized). Further, none of the included studies had a non-exercise control group to compare results, and only half had a non-pain control group. Not including a non-exercise control group fails to account for the social interaction of exercise and including a non-pain control group helps clarify whether the observed changes in brain function simply reflect the uptake of a new lifestyle behaviour or have implications for CP.

Sex differences were not analyzed in any studies included in the present review. Among studies, there was an uneven distribution of participant sex, as five included only female participants, and the other five included both sexes but with a greater percentage of females. Future research on exercise and CP should aim to achieve sample sizes large enough to assess sex differences. As previously stated, one group of authors used the same participants/dataset to produce two manuscripts. Previous fMRI research has completed multiple analyses of the same participants. Such an approach likely reflects a desire to maximize the dataset, given the cost associated with fMRI collection. However, including both manuscripts means that one cohort is reported twice within our review of 10 studies. Our review may have included more studies if we expanded our inclusion criteria to those experiencing CP as a secondary diagnosis; that is, individuals who experience pain as a result of being diagnosed with spinal cord injury, multiple sclerosis, traumatic brain injury, or some other chronic condition. The included 10 studies demonstrated varying degrees of quality, as per our bias assessment, and focused on various regions of interest. We included studies that measured brain activity and connectivity and grouped them under “brain function.” Had studies been more numerous, activity and connectivity could have been assessed in independent reviews; this further supports the need for more research exploring brain physiology in CP.

The authors of the original studies used their own preferred methods of collecting, processing, and analyzing functional imaging data**.** Recently, there has been a movement towards standardizing image collection (Duchesne et al., 2019), folder organization (Gorgolewski et al., 2016), and pre-processing (Esteban et al., 2019), but these are simply suggestions. Additionally, there is no agreed-upon method for analyzing imaging results, and as previously demonstrated, researchers can take very different approaches to answer the exact same question (Botvinik-Nezer et al., 2019). Taken together, this prevented us from conducting a meta-analysis. Correlation analysis between changes in brain function and clinical outcomes post-intervention was not conducted in any studies in this review. As a result, we reframed from exploring such outcomes.

Despite such shortcomings, our findings are encouraging given the consistency of regions altered post-exercise, regions that previous research, beyond the field of exercise, has demonstrated to be implicated in pain (Yang and Chang, 2019 Jun 26, Rusbridge, 2020, Ayoub et al., 2019, Čeko et al., 2020, Alshelh et al., 2018, Seminowicz and Moayedi, 2017). Further, included studies demonstrated an improvement in pain perception and/or quality of life post-intervention. Although the effectiveness of different exercise modalities in treating people with CP was not a focus of this review, Tai Chi and resistance training were common modalities. Future exercise interventions should aim to determine the mechanism(s) governing changes in brain function of people with CP, if it differs by exercise modality, and which exercise modality and parameters are most efficacious for a specific type of CP (El-Sayes et al., 2019); this is particularly relevant given that the present review identified 12 weeks as a threshold for altering brain function.

## 5 Conclusion

This systematic review investigated the effects of exercise on brain function (connectivity and activity) and pain perception/quality of life in people with CP. We observed that exercise (Tai Chi, resistance, and/or aerobic training) promoted upregulation between regions of the cortico-limbic pathway, as well as the default-mode network. We also observed downregulation of the DLPFC post-exercise. These changes occurred simultaneously with improvements in pain perception/quality of life. Such findings suggest that exercise may help restore or *normalize* brain function in key cortical regions of CP, with downstream implications for behavioural outcomes (i.e., pain experience and quality of life), after an exercise intervention ≥ 12 weeks.

## Declaration of Competing Interest

The authors declare that they have no known competing financial interests or personal relationships that could have appeared to influence the work reported in this paper.

## Data Availability

Systematic Review: therefore, secondary data
