# Supplementary material for: Exercise training augments brain function and reduces pain perception in adults with chronic pain: A systematic review of intervention studies
Source: Neurobiol Pain. 2023 Apr 20;13:100129. doi: 10.1016/j.ynpai.2023.100129 (PMC10189552; doi:10.1016/j.ynpai.2023.100129)
Supplement: Supplementary Data 1 [file mmc1.docx]

Supplementary Material A: Prospero pre-registration document

Supplemental Material B: Search Terms for Pubmed, EMBASE, AMED, and CINAHL.

| **Main Concept** | **Subject Heading** | | | | **Keyword in Title or Abstract** |
| --- | --- | --- | --- | --- | --- |
|  | PubMed | EMBASE | AMED | CINAHL |  |
| **Functional Neuroimaging** | “Functional neuroimaging”  “Magnetic resonance Imaging”  “Near-Infrared spectroscopy” | exp “Functional near-infrared spectroscopy”  exp “Functional magnetic resonance imaging” | exp “Magnetic resonance imaging” | exp “Magnetic resonance imaging”  Near-Infrared spectroscopy | “fMRI”  “Functional magnetic resonance imaging”  “fNIRS”  “Functional near-infrared spectroscopy” |
| **Exercise** | “Exercise therapy” | exp “Kinesiotherapy” | exp “Exercise”  exp “Exercise therapy” | exp “Exercise”  exp “Therapeutic exercise” | “Exercis*” |
| **Pain** | “Pain”  “Pain management”  “Pain perception”  “Pain measurement”  “Fibromyalgia” | exp “Pain”  exp “Nociception”  exp “Pain measurement”  exp “Fibromyalgia” | exp “Pain”  exp “Pain measurement”  exp “Fibromyalgia” | exp “Pain”  “Pain management”  “Pain Measurement”  exp “Nociceptive Pain”  “Fibromyalgia” | “Pain”  “Nocicept* “ |

Note: fMRI = Functional magnetic resonance imaging, fNRIS = Functional near-infrared spectroscopy, exp= explode. Shading used to separate concepts.

Supplementary Material C: Search strategy for Pubmed, EMBASE, AMED, and CINAHL.

**USING PUBMED DATABASE**

1 (("Functional neuroimaging"[MeSH Terms]) OR ("Magnetic resonance Imaging"[MeSH Terms])) OR (Near-Infrared spectroscopy[MeSH Terms])

2 (((fMRI[Title/Abstract]) OR ("Functional magnetic resonance imaging"[Title/Abstract])) OR (fNIRS[Title/Abstract])) OR ("Functional near-infrared spectroscopy"[Title/Abstract])

3 #1 or #2

4 Exercise[MeSH Terms]) OR ("Exercise therapy"[MeSH Terms])

5 Exercis*[Title/Abstract]

6 #4 or #5

7 ((((Pain[MeSH Terms]) OR ("Pain management"[MeSH Terms])) OR ("Pain perception"[MeSH Terms])) OR ("Pain measurement"[MeSH Terms])) OR (Fibromyalgia[MeSH Terms])

8 Pain[Title/Abstract]) OR (Nocicept*[Title/Abstract])

9 #7 or #8

10 #3 and #6 and #9

**USING EMBASE DATABASE**

1 exp " Functional near-infrared spectroscopy"/

2 exp "Functional magnetic resonance imaging"/

3 fMRI.ti. or fMRI.ab. or "Functional magnetic resonance imaging".ti. or "Functional magnetic resonance imaging".ab. or fNIRS.ti. or fNIRS.ab. or "Functional near-infrared spectroscopy".ti. or "Functional near-infrared spectroscopy".ab.

4 1 or 2 or 3

5 exp Exercise/

6 exp Kinesiotherapy/

7 Exercis*.ti. or Exercis*.ab.

8 5 or 6 or 7

9 exp Pain/

10 exp Nociception/

11 exp "Pain measurement"/

12 exp Fibromyalgia/

13 Pain.ti. or Pain.ab. or Nocicept*.ti. or Nocicept*.ab.

14 9 or 10 or 11 or 12 or 13

15 4 and 8 and 14

**USING AMED DATABASE**

1 exp Magnetic resonance imaging/

2 fMRI.ti. or fMRI.ab. or "Functional magnetic resonance imaging".ti. or "Functional magnetic resonance imaging".ab. or fNIRS.ti. or fNIRS.ab. or "Functional near-infrared spectroscopy".ti. or "Functional near-infrared spectroscopy".ab.

3 1 or 2

4 exp Exercise/

5 exp "Exercise therapy"/

6 Exercis*.ti. or Exercis*.ab.

7 4 or 5 or 6

8 exp Pain/

9 exp "Pain measurement"/

10 exp Fibromyalgia/

11 Pain.ti. or Pain.ab. or Nocicept*.ti. or Nocicept*.ab.

12 8 or 9 or 10 or 11

13 3 and 7 and 12

**USING CINAHL DATABASE**

S1 (MH "Magnetic Resonance Imaging+") OR (MH "Spectroscopy, Near-Infrared")

S2 "fMRI" OR ""Functional magnetic resonance imaging"" OR "fNIRS" OR ""Functional near-infrared spectroscopy""

S3 S1 OR S2

S4 (MH "Exercise+") OR (MH "Therapeutic Exercise+")

S5 ""Exercis*""

S6 S4ORS5

S7 (MH "Pain+") OR (MH "Pain Management") OR (MH "Pain Measurement") OR (MH "Nociceptive Pain+") OR (MH "Fibromyalgia")

S8 "Pain" OR "Nocicept*"

S9 "Pain" OR "Nocicept*"

S10 S7 OR S8 OR S9

S11 (S7 OR S8 OR S9) AND (S3 AND S6 AND S10)

Supplementary Material D: Detailed quality assessment of studies with a control group.

| **Quality Assessment Tool for Controlled Intervention Studies** | **Flodin et al, 2015 (34)** | **Kong et al, 2021 (35)** | **Lofgren et al, 2023 (42)** | **Martinsen et al, 2018 (38)** | **Micolos et al, 2014 (39)** | **Van de Winckel, 2022 (43)** |
| --- | --- | --- | --- | --- | --- | --- |
| 1. Was the study described as randomized, a randomized trial, a randomized clinical trial, or an RCT? | No | No | Yes | No | No | Yes |
| 2. Was the method of randomization adequate (i.e., use of randomly generated assignment)? | NA | NA | Yes | NA | NA | Yes |
| 3. Was the treatment allocation concealed (so that assignments could not be predicted)? | NA | NA | Yes | NA | NA | Yes |
| 4. Were study participants and providers blinded to treatment group assignment? | NR | NR | No | NR | NR | No |
| 5. Were the people assessing the outcomes blinded to the participants' group assignments? | NR | NR | Yes | Yes | NR | Yes |
| 6. Were the groups similar at baseline on important characteristics that could affect outcomes (e.g., demographics, risk factors, co-morbid conditions)? | NR | Yes | NR | Yes | NR | NR |
| 7. Was the overall drop-out rate from the study at endpoint 20% or lower of the number allocated to treatment? | No | No | No | No | NR | NR |
| 8. Was the differential drop-out rate (between treatment groups) at endpoint 15 percentage points or lower? | No | NR | Yes | Yes | NR | NR |
| 9. Was there high adherence to the intervention protocols for each treatment group? | NR | NR | NR | NR | NR | Yes |
| 10. Were other interventions avoided or similar in the groups (e.g., similar background treatments)? | NR | Yes | NR | NR | NR | NR |
| 11. Were outcomes assessed using valid and reliable measures, implemented consistently across all study participants? | Yes | Yes | Yes | Yes | Yes | Yes |
| 12. Did the authors report that the sample size was sufficiently large to be able to detect a difference in the main outcome between groups with at least 80% power? | NR | No | No | No | No | No |
| 13. Were outcomes reported or subgroups analyzed prespecified (i.e., identified before analyses were conducted)? | Yes | Yes | Yes | No | No | Yes |
| 14. Were all randomized participants analyzed in the group to which they were originally assigned, i.e., did they use an intention-to-treat analysis? | NA | NA | NR | Yes | Yes | NR |

Note: NA= not applicable, NR= not reported.

Supplementary Material E: Detailed quality assessment of studies with no control group.

| **Quality Assessment Tool for Before-After (Pre-Post) Studies with No Control Group** | **Ozuturko et al, 2020 (41)** | **Shen et al, 2021 (40)** | **Liu et al, 2019ab  (36, 37)** |
| --- | --- | --- | --- |
| 1. Was the study question or objective clearly stated? | Yes | Yes | Yes |
| 2. Were eligibility/selection criteria for the study population prespecified and clearly described? | Yes | Yes | Yes |
| 3. Were the participants in the study representative of those who would be eligible for the test/service/intervention in the general or clinical population of interest? | Yes | Yes | Yes |
| 4. Were all eligible participants that met the prespecified entry criteria enrolled? | No | Yes | Yes |
| 5. Was the sample size sufficiently large to provide confidence in the findings? | No | No | No |
| 6. Was the test/service/intervention clearly described and delivered consistently across the study population? | Yes | No | Yes |
| 7. Were the outcome measures prespecified, clearly defined, valid, reliable, and assessed consistently across all study participants? | Yes | Yes | Yes |
| 8. Were the people assessing the outcomes blinded to the participants' exposures/interventions? | NR | NR | Yes |
| 9. Was the loss to follow-up after baseline 20% or less? Were those lost to follow-up accounted for in the analysis? | NR | No | No |
| 10. Did the statistical methods examine changes in outcome measures from before to after the intervention? Were statistical tests done that provided p values for the pre-to-post changes? | Yes | Yes | Yes |
| 11. Were outcome measures of interest taken multiple times before the intervention and multiple times after the intervention (i.e., did they use an interrupted time-series design)? | No | No | No |
| 12. If the intervention was conducted at a group level (e.g., a whole hospital, a community, etc.) did the statistical analysis take into account the use of individual-level data to determine effects at the group level? | unclear | unclear | unclear |

Note: NR= not reported.

Supplemental Material F: Detailed brain connectivity measured post-intervention via functional magnetic resonance imaging (fMRI) in included studies.

| **Study ID (Author, Year)** | **Threshold** | **Type of Exercise** | **Change** | **Seed → Region of Interest** | | | **Change in Connectivity** |
| --- | --- | --- | --- | --- | --- | --- | --- |
|  |  |  |  |  | | |  |
| Flodin et al, 2015 (34) | *p*<0.05 uncorrected, corrected *p*<0.0083 | I: Resistance training C: Healthy controls | I > C | Right anterior insula | → | Left primary sensory motor areas | ↑ |
|  |  |  |  | Right supramarginal gyrus |  | Primary sensory motor areas Left inferior prefrontal cortex |  |
| Kong et al, 2021 (35) | *p*<0.05 uncorrected | I: Tai Chi  C: Healthy controls | I: Post > Pre | Medial hypothalamus | → | Left amygdala Right anterior cingulate cortex  Cerebellum Right dorsolateral prefrontal cortex  Operculum Thalamus Occipital Area | ↑ |
|  |  |  |  | Lateral hypothalamus |  | Bilateral right anterior cingulate cortex Medial prefrontal cortex  Left posterior cingulate cortex  Middle/superior temporal gyrus |  |
| Liu et al, 2019a (36) | *p*<0.05 uncorrected | I1: Baduanjin I2: Tai Chi I3: Aerobic training C: Health education | I1, I2, I3 > C  I2, 13 > C | Dorsolateral prefrontal cortex | → | Anterior cingulate cortex  Left medial prefrontal cortex | ↑ |
|  |  |  | I1, I2, I3 > C  I2, 13 > C | Dorsolateral prefrontal cortex |  | Supplementary motor area  Temporoparietal junction  Right frontal operculum Putamen Superior frontal gyrus Prefrontal/precentral gyrus Insula | ↓ |
| Liu et al, 2019b (37) | *p*<0.05 uncorrected | I1: Baduanjin I2: Tai Chi I3: Aerobic training C: Health education | I1, I2, I3 > C I1, I2 > C | Periaqueductal gray | → | Medial orbital prefrontal cortex  Anterior cingulate cortex  Right angular gyrus Middle temporal gyrus Occipital fusiform gyrus Right nucleus accumbens | ↑ |
|  |  |  | I1, I2, I3 > C I1, I2 > C | Periaqueductal gray |  | Medial orbital prefrontal cortex  Middle temporal gyrus | ↓ |
|  |  |  | I1, I3 >C | Ventral tegmental area |  | Left posterior cingulate cortex  Precuneus Lingual gyrus Thalamus Occipital fusiform gyrus | ↑ |
|  |  |  | I1, I2, I3 > C I1, I3 > C I1, I2 > C | Ventral tegmental area |  | Superior parietal lobule  Right medial orbital prefrontal cortex  Left anterior cingulate cortex | ↓ |
| Lofgren et al, 2023 (42) | p< 0.001 uncorrected, corrected p<0.05 | I: EIH C: Healthy controls | I: Post> Pre (FM group) | Caudate | → | Left inferior occipital lobe | ↑§ |
| Shen et al, 2021(40) | *p*<0.05 corrected | I: Tai Chi C: NA | I: Post > Pre | Bilateral mPFC | → | Amygdala | NC |

Note: (§) = measured during application of pain stimuli. Shading used to separate studies.

Supplemental Material G: Detailed brain activity measured post-intervention via functional magnetic resonance imaging (fMRI) and functional near infrared spectroscopy (fNIRS) in included studies.

| **Study ID (Author, Year)** | **Threshold** | **Type of Exercise** | **Change** | **Region of Interest** | **Change in Activity** |
| --- | --- | --- | --- | --- | --- |
| Lofgren et al, 2023 (42) | p< 0.001 uncorrected, corrected p<0.05 | I: EIH C: Healthy controls | I & C: Post> Pre | Left caudate  Dorsolateral prefrontal cortex | ↑§ |
| Martinsen et al, 2018 (38) | *p*<0.005 uncorrected | I: Resistance training C: Healthy controls | I > C | Cerebellum Putamen | ↑ |
|  |  |  | I: Post > Pre | Bilateral amygdala Right temporal pole |  |
| Micalos et al, 2014 (39) | *p*<0.05 corrected | I: Aerobic exercise training  C: Healthy controls | I > C | Superior temporal gyrus  Thalamus  Caudate | NC§ |
| OzturkO et al, 2021(41) | *p*<0.05 uncorrected | I: Resistance and flexibility training C: NA | I: Post>Pre | Dorsolateral prefrontal cortex | ↓§ |
| Van de Winckel et al, 2023 (43) |  | I1: Qigong I2: Whole body training | I1: Post > Pre | Parietal operculum Angular gyrus Supramarginal gyrus Precentral gyrus | ↑ |

Note: (§) = measured during application of pain stimuli. Shading used to separate studies.

Supplementary Material H: Detailed pain perception and quality of life outcome changes measured post-intervention via self-reported questionnaires.

| **Author & Year** | **Subjective Measure** | **Results (mean SD)** | | **Change** | ***p*-value** | |
| --- | --- | --- | --- | --- | --- | --- |
| Flodin et al, 2015 (34) | SF36BP  FIQ | Pre: 37.00 (9.70)  Post: 37.07 (11.8)  Pre: 60.8 (11.8)  Post: 53.3 (29.5) |  | No significant change    Improvement | | 0.040 |
| Kong et al, 2021 (35) | FIQR  BDI-II | Pre: 45.9 (7.6) Post: 36.3 (20.3)  Pre: 17.7 (9.3)  Post: 10.8 (9.2) | | Improvement  Improvement | | <0.001  <0.003 |
| Liu et al, 2019a (36) | KOOS pain | Post-pre:  I1:10.02 (12.22)  I2:10.22 (9.44)  I3: 7.37+/- 6.93 | | Improvement | | <0.01 |
| Liu et al, 2019b (37) | KOOS pain | Post-pre:  I1: 10.0(12.2)  I2: 10.2(9.4)  I3: 7.4(6.9) | | Improvement | | <0.01 |
| Lofgren et al, 2023 (42) | VAS  FIQ  HADS-D  HAD-A  Pressure rating | Pre-post: I1: 23  I2: 0.5   I1: 3.6  I2: 8.5  I1: 2.0 I2: 0.5  I: 1.0 I2: 1.5  Specific values NR | | Improvement  No significant change  Improvement  Improvement  Improvement  No significant change  No significant change  No significant change  No significant change | | 0.001   0.002 0.025   0.010 |
| Shen et al, 2021(40) | VAS  WOMAC | Specific values NR  Specific values NR | | Improvement  Improvement | | 0.018  0.021 |
| Martinsen et al, 2018 (38) | VAS  SF-36    FIQ | Pre: 44.6 (17.5)  Post: 41.1 (21.0)  SF-36-PCS pre-I: 33.2 (11.7)  SF-36-PCS post-I: 36.6 (16.4)  SF-36-MCS pre-I: 35.3 (10.0) SF-36-MCS post-I: 38.2 (11.8)  Pre: 59.9 (16.0)  Post: 54.7 (19.8) | | Improvement  Improvement  Improvement | | 0.001  0.001  0.001 |
| OzturkO et al, 2021 (41) | VAS  WOMAC | Pre: 6.29 (1.49) Post: 3.00 (1.84)  Post-Pre: 13.8 (8.61) | | Improvement  Improvement | | 0.001  0.001 |
| Micalos et al, 2014 (39) | SF-36  MPQ  Pressure rating | Pre: 29.6 (15.3)  Pre: 19.2 (11.7)  Pre: 4 Post: ~4.5 | | Not reported  Not reported  No significant change | |  |
| Van de Winckle et al, 2022 (43) | NPRS ^o^ | Pre-post I1: 3.86 I2: 5.06 | | Improvement Improvement | | <0.0002 <0.0001 |
|  |  |  | |  | |  |

Note: SF36= Short Form Health Survey, SF36BP = Bodily Pain Subscale of Short Form Health Survey, FIQ = Fibromyalgia Impact Questionnaire, FIQR=Fibromyalgia Impact Questionnaire Revised, BDI-II = Beck Depression Inventory, KOOS = Knee Injury and Osteoarthritis Outcome Score, VAS = Visual Analog Scale, HADS = Hospital Anxiety and Depression Scale, MPQ = McGill pain Questionnaire, WOMAC = Western Ontario and McMaster Universities Osteoarthritis Index, NPRS= Numerical pain rating scale, ^o^= additional questionnaires used. Shading used to separate studies.
